# A longitudinal study monitoring the quality of life in a national cohort of older adults in Chile before and during the COVID-19 outbreak

**DOI:** 10.1186/s12877-021-02110-3

**Published:** 2021-02-26

**Authors:** M. Soledad Herrera, Raúl Elgueta, M. Beatriz Fernández, Claudia Giacoman, Daniella Leal, Pío Marshall, Miriam Rubio, Felipe Bustamante

**Affiliations:** 1 Instituto de Sociología (Department of Sociology), Pontificia Universidad Católica de Chile, Av.Vicuña Mackenna 4860 Macul, Santiago, Chile; 2 Instituto de Estudios Avanzados (Institute of Advanced Studies), Universidad de Santiago de Chile, Santiago, Chile; 3 Escuela de Enfermería (Nursing School), Pontificia Universidad Católica de Chile, Santiago, Chile

**Keywords:** COVID-19, Confinement, depressive symptoms, resilience, connectedness, loneliness, Health problems, Smartphone

## Abstract

**Background:**

Confinement during the COVID-19 pandemic has placed great stress on older adults, which may be affecting their quality of life. Thus, this study aims to describe the changes in mental and physical health, isolation and loneliness, residence and socioeconomic resources in a national cohort of Chilean older adults before and during the COVID-19 outbreak. It also analyzes the changes in depressive symptoms by changes in the other quality of life indicators before and during the COVID-19 outbreak. Possible methodological biases of telephone surveys in older adults living in non-developed countries are also discussed.

**Methods:**

Between June and September 2020, a random subsample of 720 people who had participated in the face-to-face V National Survey on Quality of Life in Older Adults in Chile conducted at the end of 2019 was followed up by telephone. Descriptive bivariate analyses were performed using t-test and non-parametric tests for independent variables, comparing the baseline sample with the current 2020 follow-up sample during the peak of the pandemic outbreak in Latin America. Furthermore, descriptive bivariate analysis through t-test and non-parametric test for paired samples compared the follow-up subsample at baseline with the not-included sample, examining possible biases of the telephone interview compared with the face-to-face interview.

**Results:**

In the panel, there was no variation in self-rated health. The health symptoms that worsened were memory, stomach, and mood problems. Depressive symptoms and anxiety increased; similarly, smartphone users, social contacts, intergenerational co-residence and resilience increased. The telephone follow-up sample had a higher educational level and greater smartphone use than those not included in the subsample.

**Conclusions:**

Although some physical and mental health indicators have worsened during the pandemic, older adults mobilized resources that could allow them to maintain their quality of life, such as improved resilience. Thus, these findings can guide future research and the development of efficient strategies to improve these resources among older adults to ensure wellbeing.

**Supplementary Information:**

The online version contains supplementary material available at 10.1186/s12877-021-02110-3.

## Introduction

Chile is a middle-developed Latin American country that has a rapidly aging population. In 2019, the population aged 65 and above was more than 2,260,222 people, corresponding to 11.9% of the Chilean population. By 2035, this group is projected to reach 18.9% of the population, surpassing the population aged 0–14 years by 16.1% [1].

Villalobos et al. [2] summarized the key events of the COVID-19 pandemic in Chile up to June 2020. The first confirmed case of COVID-19 was on March 3, 2020, and 15 days later, a state of constitutional exception was declared due to the national catastrophe. This gave the government the power to restrict freedom of movement and association, thereby establishing a mandatory home quarantine for people older than 80 years (changed to 75 years on May 15) through the restriction of visits to long-term care centers and the closing of all daily centers, clubs, and organizations for older adults. Many health checks, scheduled procedures, and surgeries were also restricted and suspended. A dynamic quarantine system was established, based on sanitary criteria, with a special permit needed for food and other belongings to be supplied to older adults, although this was restricted to only twice a week in some areas. By the end of October 2020, half a million of Chilean people had been infected, and almost fourteen thousand people had died from COVID-19 (https://paho-covid19-response-who.hub.arcgis.com/).

Media channels pointed out that older adults would be the most affected by the pandemic, not only because they are the group with the highest mortality risk from the disease [3], but also because of the conditions of forced or voluntary confinement, which would have physical and mental health consequences [4, 5]. For example, confinement restrictions were the main stressful event mentioned in an online survey of 825 U.S. adults aged 60 years and older [6].

Some studies conducted during the COVID-19 pandemic have reported worsening quality of life indicators among older adults. In a cross-sectional survey conducted in Italy, France, and Spain, Arpino et al. [7] found that approximately 50% of individuals aged 50+ felt sad or depressed more often than usual during the lockdown. Furthermore, in a panel study of a Dutch older sample, Van Tilburg et al. [8] found that social and emotional loneliness increased during a seven-month period that began before the pandemic and ended during confinement due to COVID-19. They also found an increase in depression and anxiety. Similarly, Krendl and Perry [9] found that older adults had higher levels of depression and loneliness during the COVID-19 outbreak than before the pandemic, according to a follow-up study of older adults in the United States. Furthermore, perceived closeness to network members, but not social engagement, moderated the relationship between loneliness and depression.

However, other studies have found no significant changes in the wellbeing of older adults during the pandemic. To illustrate, Whitehead and Torossian [6] found no correlation between confinement, as one of the most stressful events of the pandemic, and three indicators of psychological wellbeing: perceived stress, negative affect, and positive affect. Kivi et al. [10] concluded that COVID-19 had exerted few adverse effects on wellbeing among older adults in Sweden. Instead, most people rated their wellbeing just as high or even higher than they did in previous years.

Studies have found that for the population aged 75 years and above, the lockdown can also have consequences on their image, intensifying the occurrence of ageism [11–15] . Furthermore, during the confinement period, older adults were restricted from actively participating in various aspects of everyday life, such as through working and other means of contributing to society, thus highlighting older adults’ view as being an inactive segment of society in need of help. In summary, many studies show the risks of ageism on the wellbeing and health of older adults [15–17].

In a literature review, Tripathy [18] noted a scarcity of research articles focused on older adults. The concern for older adults’ mental health during the pandemic has been mostly expressed in letters to health journal editors. Furthermore, most studies monitoring mental health indicators during the pandemic have been conducted with the younger population and have used online surveys [19–21]. Although some research has included older populations, the majority have been cross-sectional or longitudinal studies that began during the pandemic [7, 22–24].

In this context, the present study aims to monitor some quality of life indicators in a representative national cohort of older adults in Chile from during and before the pandemic COVID-19.

The first contribution of the present study is the longitudinal (panel) design with a baseline measure just before the pandemic in Chile. To date, our research team found only four published studies explicitly conducted with older adults based on a panel sample with a baseline before the pandemic [8–10, 25]. Like the present study, the variables included social networks, loneliness, anxiety, depression, self-rated health, and financial satisfaction. Nevertheless, they were placed only in developed countries.

So, the second major novelty of the present study is that it is the first to report comparative results using a set of quality of life indicators in a national panel sample of older adults in a Latin American middle-income country as Chile.

The third contribution would be to discuss possible methodological biases of telephone surveys in older adults living in non-developed.

## Methods

### Participants

This study is based on data from the V National Survey on Quality of Life in Older Adults (Quality of Life Survey - QLS) and the COVID-19 Panel Survey. The first survey was conducted face-to-face with a representative sample of 2132 60-year-old adults living in the community during November 28, 2019, and January 19, 2020. The respondents were selected by systematic randomization of blocks, private dwellings, and older adults inside their selected dwellings. Furthermore, people over 80 years were oversampled through compulsory inclusion if they lived in the selected dwellings. The study only included people who were not suspected of having cognitive deterioration screened with the Mini-Cog [26, 27].

A computer-assisted standardized telephone interview was used to administer the COVID-19 Panel Survey to a random sample of 721 older adults (60+ years) selected from the cases that participated in the first wave of the QLS in 2019. The sampling frame included participants with valid telephone numbers who agreed to be interviewed again (69.09% of the total sample). Furthermore, individuals living alone in 2019 were included in the selected sample (16.90% of the sampling frame). The COVID-19 sample was selected by utilizing systematic random sampling from the eligible cases remaining, ordered by gender and age. The COVID-19 Panel Survey would include two additional waves from October to December 2020 and January to March 2021.

All respondents gave their informed consent. The project was subject to ethical review at all stages and was approved by the Ethical Committee of the Pontificia Universidad Católica de Chile (ID Protocol: 200514003, May 27, 2020). 

### Measures

#### Physical and mental health measures

Self-rated health was evaluated with the following question: Do you consider your health: Excellent, good, regular, or bad? which was dichotomized into bad/regular and excellent/good.

Since 2007, the QLS has regularly measured thirteen health symptoms in Chile: back pain, knees, hips, or other joint pains; choking when walking or chest pain; persistent cough or shortness of breath; headache; memory problems; swollen legs; fall or fracture; dizziness or fainting; stomach or bowel problems including constipation, gas, and diarrhea; incontinence or involuntary loss of urine; feeling down; alcohol abuse; and illicit drug use [28]. Thus, it is considered a valid measure and was utilized in the current study to measure older adults’ health symptoms.

The Patient Health Questionnaire (PHQ-9) for depressive symptoms [29] consisted of nine items that were scored as follows: 0 = never, 1 = several days, 2 = more than half of the days, 3 = almost every day; the total scores ranged from 0 to 27. This questionnaire has been validated in Chile in a sample of adults from primary care centers [30]. It has shown high internal consistency, significant factor loadings, 80% sensitivity, and 77% specificity for a cut-off score of 7 for major depressive disorder.

The Geriatric Anxiety Inventory - Short Form (GAI-SF) [31] was composed of five dichotomous items, which ranged on a scale from 0 to 5, with a suggested cut off point of > = 3 for the detection of anxiety, a sensitivity of 75%, and a specificity of 87 and 86% for correctly classified individuals; Cronbach’s alpha was 0.81 for this scale.

The Brief Resilient Coping Scale (BRCS) had four items with five response alternatives, ranging from 0 to 16 [32]. It has already been adapted and validated in Spain’s older adult population [33], and has shown adequate internal consistency and positive correlations with coping resources and psychological wellbeing.

#### Social and economic measures

The Abbreviated Brief Lubben Social Network Scale for isolation LSNS-6 is an instrument designed to measure social isolation in older adults, based on the amount and frequency of social contact they have with their family and friends and the perception of social support and closeness received from these sources. It consists of six Likert-type questions with six response options ranging from 0 = *none* to 5 = *nine or more.* So, the range is from 0 (total isolation) to 30 (no isolation). The scale demonstrated high internal consistency levels, stable factor structures, and high correlations with criterion variables; score less than 12 indicates social isolation [34].

The revised UCLA Three-Item Loneliness Scale [35] had three items with three possible responses from 0 = *hardly ever*, 1 = *some of the time*, or 2 = *often*, with total scores ranged between 0 and 6. As the scale is positively skewed, it was dichotomized in those who reported never being lonely (score of 3) and those who reported being lonely some or all of the time (scores greater than 3) [36].

For the family structure, questions were asked to determine if the respondents lived alone or together with others, and if they lived with their children and/or grandchildren.

To determine whether participants were smartphone users, we inferred the response from the questions about the respondent’s activities via their mobile phone: chat, video calls, information search, and online procedures. If the respondent participated in at least one of these activities, they were codified as a smartphone user.

For working, the question was “during the last month and not counting housework at home, have you done any paid work, even for a few hours?” (yes/no). For the perception of income sufficiency, the following question was asked: Is the money that you have enough to satisfy your needs? with options for answers including Yes, more than enough; Yes, just enough; or No.

#### Socio-demographic variables

Socio-demographic variables included gender, age (60–74 or 75 and over), education (primary: eight or fewer years of education, secondary: from nine to 12 years of education, higher: at least 1 year of university or professional studies), marital status (single, married/cohabitant, separated/divorced, widowed).

### Statistical analyses

The first analysis included a comparison of the COVID-19 sample before the pandemic (November 2019–January 2020) and during the peak of the first wave of COVID-19 in Chile (June–September 2020). All variables likely to vary between the two waves were included, excluding variables relatively fixed in time such as gender, age, marital status, and educational level. A detailed analysis of the differences in each of the items of the scales was also conducted. Paired t-tests of means were used for the scales and paired non-parametric tests for the dichotomous variables. Stata 14.2 software was used to conduct all analyses.

To jointly analyze how these changes affect the quality of life of older adults, change variables were calculated on the measured scales (for each individual, it was calculated as the value of a variable during the pandemic minus the value of the same variable before the pandemic). A multiple regression model was calculated on the changes in depressive symptoms, predicted by changes in the scales of resilience, isolation, loneliness and sum of health problems.

The final analysis included a comparison of the baseline data for the COVID-19 sample with two other samples: i) the cases not selected for this COVID-19 study (QLS 2019/1 sample), either because they did not have a valid telephone number to be able to contact them or because we determined that we would have a sufficient sample size without including them; ii) the selected cases that could not be contacted or who refused to participate in the study (QLS 2019/2 sample). This analysis allowed us to infer possible biases of a telephone survey administered in a country where approximately half of older adults have complete or incomplete primary education. The statistical differences between these three samples were tested by conducting a *t*-test of means and a *z*-test of proportions for independent samples.

## Results

### Main differences in quality of life indicators before and during the COVID-19 outbreak

Table 1 and Table 2 compare the differences over time observed in the COVID-19 sample before the pandemic and during the first confinement peak in Chile. Table 1 includes the physical and mental indicators, and Table 2, the social and economic indicators.

**Table 1:** Changes in Indicators of Physical and Mental Health between Baseline and First Follow-up

		COVID-19 sample in the 2019 baseline	First COVID-19 sample follow-up	p (two-tailed)
Health	% Bad/ regular	55.49	54.58	.72
Health symptoms Scale (0–13)	Mean	2.82	2.96	.15
Back pain, knees, hips, or other joint pains	% Yes	63.09	64.77	.50
Choking when walking or chest pain	% Yes	16.48	17.05	.76
Persistent cough or shortness of breath	% Yes	14.26	11.65	.14
Headache	% Yes	34.91	29.26*	.02
Memory problems	% Yes	27.06	38.19***	.00
Swollen legs	% Yes	33.70	20.80***	.00
Fall or fracture	% Yes	10.05	3.74***	.00
Dizziness or fainting	% Yes	16.64	12.48*	.02
Stomach or bowel problems including constipation, gas, and diarrhea	% Yes	21.44	33.47***	.00
Incontinence or involuntary loss of urine	% Yes	12.98	21.52***	.00
Feeling down	% Yes	34.50	44.58***	.00
Alcohol abuse	% Yes	3.32	9.84***	.00
Illicit drug use	% Yes	5.68	10.81***	.00
PHQ-9 for depressive symptoms (0–27)	Mean	4.25	5.05***	.00
Dichotomous PHQ-9 > =7 (30)	% suspicious depression	23.80	30.18**	.00
Little interest or pleasure in doing things	% More than half the days	9.84	14.42**	.00
Feeling down, depressed, or hopeless	% More than half the days	14.97	20.38**	.00
Trouble falling or staying asleep, or sleeping too much	% More than half the days	17.19	28.71***	.00
Feeling tired or having little energy	% More than half the days	18.16	20.24	.31
Poor appetite or overeating	% More than half the days	10.81	16.08**	.00
Feeling bad about yourself — or that you are a failure or have let yourself or your family down	% More than half the days	7.90	9.29	.34
Trouble concentrating on things, such as reading the newspaper or watching television	% More than half the days	7.21	7.48	.84
Moving or speaking so slowly that other people could have noticed? Or the opposite — being so fidgety or restless that you have been moving around a lot more than usual	% More than half the days	3.74	8.59***	.00
Thoughts that you would be better off dead or of hurting yourself in some way	% More than half the days	3.46	1.94	.07
Geriatric Anxiety Inventory GAI-SF (0–5)	Mean	2.04	2.26**	.00
Dichotomous GAI-SF > =3 (31)	% with anxiety	40.00	42.85	.27
I worry a lot of the time	% Yes	44.10	47.71	.16
Little things bother me a lot	% Yes	24.82	28.57	.10
I think of myself as a worrier	% Yes	51.17	64.07***	.00
I often feel nervous	% Yes	43.55	46.18	.31
My own thoughts often make me anxious	% Yes	36.06	39.80	.14
Brief Resilient Coping Scale (BRCS) (0–16)	Mean	11.11	13.26***	.00
I look for creative ways to alter difficult situations	% Describes very well	14.93	55.38***	.00
Regardless of what happens to me, I believe I can control my reaction to it	% Describes very well	18.15	55.64***	.00
I believe that I can grow in positive ways by dealing with difficult situations	% Describes very well	19.57	60.60***	.00
I actively look for ways to replace the losses I encounter in life	% Describes very well	17.57	58.30***	.00

*N* = 721 older adults; *p < .05, **p < .01, ***p < .001

**Table 2:** Changes in Indicators of Social and Economic Quality of Life between Baseline and First Follow-up

		COVID-19 sample in the 2019 baseline	First COVID-19 sample follow-up	p (two-tailed)
Brief Lubben Social Network Scale (0–30)	Mean	12.93	14.53***	.00
Dichotomous Brief Lubben Social Network <=12 Scale (34)	% Isolated	50.76	38.61***	.00
How many relatives do you see or hear from at least once a month?	% > =3	75.00	87.84***	.00
How many relatives do you feel at ease with that you can talk about private matters?	% > =3	49.51	50.20	.79
How many relatives do you feel close to such that you could call on them for help?	% > =3	49.72	56.52**	.00
How many friends do you see or hear from at least once a month?	% > =3	42.06	58.71***	.00
How many friends do you feel at ease with that you can talk about private matters?	% > =3	24.47	22.36	.34
How many friends do you feel close to such that you could call on them for help?	% > =3	23.88	30.72**	.00
UCLA-3 Loneliness Scale (0–6)	Mean	1.06	1.11	.49
Dichotomous UCLA-3 Loneliness Scale <=3 (36)	% Alone	43.31	47.75*	.04
How often do you feel that you lack companionship?	% Some of the time/often	37.04	42.31*	.04
How often do you feel left out?	% Some of the time/often	21.45	19.97	.48
How often do you feel isolated from others?	% Some of the time/often	21.14	20.31	.69
Live alone	% Alone	21.67	16.64*	.01
Live with children	% Yes	43.07	50.90**	.00
Live with grandchildren	% Yes	24.89	31.94**	.00
Smartphone	% User	45.90	54.78***	.00
Working	% Working	28.11	22.53*	.01
Income	% Not sufficient	34.10	29.34	.05

*N* = 721 older adults; *p < .05, **p < .01, ***p < .001

There were no statistically significant changes in the global health indicators: self-rated health and sum of health symptoms. However, there were several changes in specific health problems. For example, memory problems increased from 27.06 to 38.19%; stomach problems increased from 21.44 to 33.47%; feeling down increased from 34.50 to 44.58%. The percentages of people newly reporting these problems were 22.47, 23.15, and 24.58%, respectively (results not shown).

It should be noted that four health symptoms improved: participants reported fewer falls or fractures at follow-up (10.05 and 3.74% at baseline and follow-up, respectively), fewer headache (from 34.91 to 29.26%), less dizziness (from 16.64 to 12.48%), and fewer swollen legs (from 33.70 to 20.80%). The latter result may be due to seasonal changes in both surveys, since the first was done in summer and the second in winter. Reports of urinary incontinence, alcohol abuse and illicit drug use increased at follow-up, but the result could be due to a sensitive question effect, given that the first survey was conducted face-to-face and the second by telephone. Problems that did not change were bone pain, heart pain, respiratory pain, self-reported excessive alcohol and illegal drug use.

Depressive symptoms increased from baseline to follow-up, from an average of 4.25 to 5.05 on the PHQ-9 scale (*p* < .001), with the item concerning sleep problems more than half the days being the most affected (from 17.19 to 28.71%). Furthermore, 19.97% of people declared sleep problems during COVID-19, although they did not declare this type of problem at baseline.

Anxiety symptoms also increased, with the GAI-SF scale score increasing from 2.04 to 2.26 (*p* < .05), and the item that increased the most was “I think of myself as a worrier.” (from 51.17 to 64.07%). In addition, 26.35% of the COVID-19 sample became a person of concern during the pandemic (not shown).

The Brief Resilience Coping scale score increased from 11.11 to 13.26 (*p* < .001), and all items increased, with the highest category of each item (“describes me very well”) being the one that increased the most significantly. For example, at baseline, between 14.93 and 19.57% of people answered this category, while at follow-up, between 55.38 and 60.60% answered this category.

Regarding social indicators (Table 2), the UCLA-3 scale of loneliness did not show significant variations, although the Lubben scale of social networks increased from 12.93 to 14.53 between baseline and follow-up (*p* > .001). The percentage of people who saw or heard about three or more friends and relatives varied the most, increasing from 42.06% at baseline to 58.71% at follow-up for friends, and from 75.00 to 87.84% for relatives. The perception of support availability (“you could call on them for help”) from family members increased from 49.72 to 56.52%, and from friends from 23.88 to 30.72%. Closeness with friends and family members did not change.

Changes were observed in residential configurations, with a decrease in those who lived alone from 21.67 to 16.64%, and 10.07% of the living arrangement for the COVID-19 sample changed from living alone to accompanied in 2020 (not shown). Those who lived with children increased from 43.07 to 50.90%, and those who lived with grandchildren increased from 24.89 to 31.94%.

There were no statistically significant differences in the perception of income sufficiency at the 95% confidence level, despite the significant decrease in the proportion of people who work from 28.11 to 22.53%. Furthermore, 14.45% of people abandoned their work. In addition, the percentage of smartphone users increased from 45.90 to 54.78%, with 21.07% of new smartphone users.

### Main changes in depressive symptoms by changes in the other quality of life indicators

Table 3 contains a multiple regression analysis on changes in depressive symptoms. The increase in depressive symptoms is explained mainly by three observed changes: the increase in the sum of health problems (β = .295, *p* = .000), in the Gai-SF anxiety scale (β = .261, *p* = .000), and the UCLA-3 loneliness scale (β = .187, *p* = .000). Changes in resilience and social networks did not affect the changes in depressive symptoms. Neither did the control variables (gender, age, education, income and smartphone).

**Table 3:** Multiple regression analysis on change in depressive symptoms

	Beta Coefficient	Standardized Beta	Standard Error	P > |t|
Change in Geriatric Anxiety Scale (mean: .224; st.dev.: 1.994; range: −5 to 5)	.219	.322	.024	0.000
Change in Brief Resilient Coping Scale (mean: 2.152; st.dev.: 4.113; range: − 16 to 16)	−.020	−.062	.011	0.075
Change in Brief Lubben Social Network Scale (mean: − 1.593; st.dev.: 6.794; range: − 30 to 30)	−.005	−.026	.007	0.459
Change in UCLA-3 Loneliness Scale (mean: .047; st.dev.: 1.872; range: −6 to 6)	.150	.207	.026	0.000
Change in Health Problems (mean: .143; st.dev.: 2.676; range: −9 to 9)	.149	.295	.017	0.000
Female	−.023	−.007	.100	0.816
Age	.007	.040	.105	0.947
Age^2^	.000	−.015	.000	0.979
Secondary Education (vs. Primary)	.016	.005	.107	0.882
Superior Education (vs. Primary)	−.031	−.009	.130	0.811
Sufficient income (0–1)	.020	.006	.102	0.841
Smartphone user (0–1)	−.083	−.030	.101	0.410
Constant	−.189		3.850	0.961

R^2^ = .2229; *N* = 663

Note: the change variables are calculated as the COVID-19 value (during the peak of the COVID-19 pandemic) minus the baseline value (before the pandemic)

The variable “change in depressive symptoms” has a mean of .156, standard deviation of 1.348 and a range from −4 to 4

### Main differences between the follow-up subsample and the not-follow-up sample at baseline

Table 4 shows the comparison between the COVID-19 sample at baseline with respect to the non-selected cases (QLS 2019/1 Sample) and those that did not respond to the follow-up study (QLS 2019/2 Sample). The COVID-19 sample had a slightly higher proportion of women (69.76%) than QLS 2019/1 (65.11%) but similar to QLS 2019/2 (64.93). Also, the COVID-19 sample had a slightly lower mean age (71.59 years) than QLS 2019/1 (72.65 years) and QLS 2019/2 (72.89 years). There were no differences in marital status. The COVID-19 sample had a lower percentage of people with primary education (43.55%) than the other two samples (49.61 and 56.10% in the QLS 2019/1 and QLS 2019/2 samples, respectively).

**Table 4:** Characteristics at baseline: Comparison of COVID-19 Sample with QLS 2019/1 (not selected) and QLS 2019/2 (no response)

		Quality of Life Survey Samples for 2019^a^			Differences at Baseline^b^	
		QLS 2019/1 (not selected)	QLS 2019/2 (no response)	COVID-19 sample in the 2019 baseline	Difference between QLS 2019/1 and COVID-19 sample 2019	Difference between QLS 2019/2 and COVID-19 sample 2019
Gender	% Female	65.11*	64.93	69.76	−4.64 (*p* = .04)	−4.80 (*p* = .07)
Age	Mean	72.65**	72.89**	71.59	1.06 (*p* = .00)	1.30 (.00)
Marital status	% Widow	30.44	27.57	29.41	1.03 (*p* = .65)	−1.83 (*p* = .48)
Education	% Primary	49.61**	56.10***	43.55	6.06 (.01)	.12(*p* = .00)
Health	% Bad/regular	52.00	53.67	55.49	−3.49 (*p* = .16)	− 1.81 (*p* = .53)
Health symptoms Scale (0–13)	Mean	2.79	2.97	2.82	−.01 (*p* = .87)	.15 (*p* = .29)
PHQ-9 for depressive symptoms (0–27)	Mean	3.83	4.16	4.25	−.42 (*p* = .07)	−.09 (*p* = .72)
Geriatric Anxiety Inventory GAI-SF (0–5)	Mean	1.80**	1.95	2.04	−.23 (*p* = .00)	−.08 (*p* = .41)
Brief Resilient Coping Scale (BRCS) (0–16)	Mean	10.29***	10.33***	11.10	−.81 (*p* = .00)	−.77 (*p* = .00)
Brief Lubben Social Network Scale (0–30)	Mean	12.47	11.81**	12.93	−.45 (*p* = .13)	−1.11 (*p* = .00)
UCLA-3 Loneliness Scale (0–6)	Mean	1.22	1.16	1.06	.16 (*p* = .05)	.10 (*p* = .26)
Live alone	% Live alone	16.21**	18.61	21.67	−5.45 (*p* = .00)	−3.06(*p* = .19)
Live with children	% Yes	46.23	43.08	43.07	3.15 (*p* = .20)	0.00 (*p* = .99)
Live with grandchildren	% Yes	27.05	26.67	24.89	2.155 (*p* = .32)	1.78 (*p* = .48)
Smartphone	% User	36.10***	29.92***	45.90	−9.80 (*p* = .00)	−15.98 (*p* = .00)
Working	% Working	25.41	25.00	28.11	−2.69 (*p* = .22)	−3.11 (*p* = .22)
Income	% Not sufficient	31.69	29.97	34.10	−2.39 (*p* = .31)	−4.11 (*p* = .13)

^a^N of QLS 2019/1 sample = 903; N of QLS 2019/2 sample = 508; N of COVID-19 sample = 721

^b^Paired t-test of means for scale variables and paired non-parametric test of proportions for dichotomous variables

*p < .05, **p < .01, ***p < .001

There were no significant differences in health (self-rated health and sum of health symptoms) between the three samples. Furthermore, there were no statistically significant differences in the average scores of the PHQ-9 for depressive symptoms. The GAI-SF scale score was slightly higher (2.04) in the COVID-19 sample than in the QLS 2019/1 sample (1.80), with no statistically significant differences with QLS 2019/2 sample. The average score of the Resilience Scale was also slightly higher in the COVID-19 sample (11.10) than in the QLS 2019/1 (10.29) and QLS 2019/2 samples (10.33).

Among the social and economic variables, the Lubben Scale score was slightly higher in the COVID-19 sample (12.93) than in the QLS 2019/2 sample (11.81). However, there were no large differences with QLS 2019/1 sample (12.47). There were no differences in the UCLA-3 loneliness scale, sufficiency of income, and labor participation.

Regarding residence, the COVID-19 sample at baseline included all people who lived alone in the 2019 survey, as their frequency was low in Chile. Therefore, the COVID-19 sample had a higher percentage (21.67%) of people living alone in 2019 than the total percentage of older adults who lived alone that year (17.70%) [28]. However, there was no difference in the percentage of older adults living with children and/or grandchildren between the samples.

Also, smartphone use was higher (45.90%) in baseline than for the 2019/1 and 2019/2 samples (36.10 and 29.92%, respectively).

## Discussion

The COVID-19 pandemic has resulted in some adverse effects on quality of life, especially in older adults. As there is a scarcity of research investigating the effects of the confinement imposed by government restrictions to curb the spread of the virus, this study aimed to compare the results of a set of quality of life indicators before and during the peak of the lockdown period. Thus, this article reported the main changes in some quality of life indicators in a heterogeneous cohort of older adults in a developing Latin American country, Chile, between the end of 2019 and early January 2020 (Chilean winter, before COVID-19) and the middle of 2020 (during Chilean winter, the lockdown of the majority of the country, and the peak of the pandemic’s first wave).

Overall, there were changes in almost all indicators, although not necessarily in the direction of worsening quality of life. On the one hand, confinement resulted in great stress and concern for older adults, increasing their levels of anxiety, depressive symptoms, and other health problems such as memory problems, stomach or bowel problems, feeling down and sleeping problems. The increase in health problems and anxiety feelings was associated with increased depressive symptoms during the COVID-19 confinement. To our knowledge, this is the first study to document the association between changes in psychosocial variables and change in depressive symptoms in older people, with a starting time just before the pandemic.

On the other hand, older adults improved resilience resources and social networks. Nevertheless, it is interesting to note that there was no association between increased resilience and decreased depressive symptoms. At the cross-sectional level, the variables were correlated (Table S1). Also, there is so much evidence in the literature of the association between greater resilience and less depression [37–39]. Maybe, the increase in resilience could be a moderating or mediating variable in the relationship between other stressors and the increase in depressive symptoms. For example, between the increase in illnesses or decreased functioning and the impact on depressive symptoms [40, 41], or between isolation or loneliness and depressive symptoms [42, 43].

The number of connections with relatives and friends also augmented, probably through digital technologies, because smartphone users also increased. The reported number of social support members (Lubben Scale item: “How many relatives/friends do you feel close to such that you could call on them for help?) also increased. Furthermore, there were changes in family configurations whereby older adults lived with their children and grandchildren.

Nevertheless, there was no association between connectedness changes and changes in depressive symptoms. At the cross-sectional level, these variables were correlated (Table S1), as has been shown in other studies [44–47]. This could be because the present research did not distinguish between in-person meetings and remote connectedness as the study by Xie et al. [47]. In Chile, as in other Latin American countries, in-person relationships are meaningful, and older people are not used to digital mediated relationships. Therefore, the increase in remote contacts was not able to replace the decrease in face-to-face contacts for diminishing depressive symptoms. However, the remote contacts seem to be protecting against the increase in depressive symptoms.

One unexpected result was that the subjective loneliness did not change. These results were not entirely consistent with other longitudinal panels. In an older Dutch panel, van Tilburg et al. [8] found an increase in social and emotional loneliness.

The increase in remote social connectedness with family and friends, partly related with the increase in smartphone use and changes in residential configurations, could have moderated the possible negative impact of the pandemic lockdown on loneliness. Some studies have documented the relationship between internet use and greater satisfaction with family and friends’ contact [48].

Although the loneliness did not change significantly, there was a significant association between increased loneliness and increased depressive symptoms. In a U.S. older panel, Krendl and Perry [9] also found increased loneliness associated with increased depressive symptoms, which was moderated by social ties’ strength.

Considering the cut-off point for loneliness proposed by Perissinotto (2012), 35.40% of the sample remained not lonely, 25.25% remained lonely, 22.43% acquired loneliness during the pandemic, and 16.93% stopped feeling lonely. This represents the enormous heterogeneity in the Chilean older adult population, as reported in other studies [28, 49].

However, in a panel study in North American adults before and during the pandemic, Luchetti et al. [50] found results similar to the present study, but for a sample of adults of all ages. Furthermore, there were no significant mean-level changes in loneliness, and there was an increase in perceived support. Although they reported lower levels of loneliness in the sample of older adults compared to younger people, they were the only group that showed a slight increase in loneliness.

Van Tilburg et al. [8] also found lower mental health due to the pandemic (more anxiety and depression). Contrastingly, Kivi et al. [10] found no adverse effects on wellbeing during the pandemic’s early stage. However, as in the present study, they found that worried people reported lower wellbeing levels. The authors interpreted these results in terms of resilience and showed heterogeneity among older adults during the pandemic. Although some traumatic events are highly prevalent stressors among older adults, many individuals report high psychological wellbeing [23]. Furthermore, some studies have shown that the pandemic had a lower impact on stress, depression, and anxiety in older adults than in younger people [51, 52].

Finally, similar to the results by Röhr et al. [24], our results did not support the common ageist stereotypes of ‘the weak and vulnerable elderly’ during the pandemic. These results have confirmed that older adults have social and psychological resources that allow them to face adversity. Older people have overcome past stressful experiences that could be encouraging them to overcome the confinement problems [53, 54].

### Limitations and future research

Despite their relevance, these results should be interpreted with caution [55], since most of the studies on older adults in developed countries were online surveys, which may have representativeness biases in favor of the more educated, especially in the older adult population. Similarly, in the present sample, telephone surveys may have overrepresented more highly educated people, as other telephone surveys do [56]. Therefore, telephone studies in Chile’s older adult population may present biases in favor of the most educated and digitally connected.

The present study had the advantage of including information about the non-included sample in the follow-up study. The COVID-19 sample had more education and smartphone user experience than the non-selected cases. In another panel study of older adults in Chile, conducted through in-person interviews, there were no differences in attrition rate based on educational level [57]. One could infer that the educational differences between the COVID-19 sample at baseline and the follow-up sample are most likely due to the modality of the survey administration (by telephone) in the follow-up sample. Nevertheless, this was the only alternative during the pandemic. Furthermore, online studies in a Latin American country as Chile could practically not be conducted in older adults. This is because less than a third of older Chileans reported that they could use the internet themselves [28].

It should be noted that this study is still ongoing. Two additional measurements will be carried out on this same sample between October and December 2020 and January and March 2021. From the first descriptive analysis presented in this article, the need to continue delving into the following topics emerged. Firstly, the role of other variables that mediate the relationship between exposure to confinement and loneliness and its effect on depressive symptoms must be explored, e.g., proactive coping [58], resilience [59, 60], and residential change (living alone, living with others, intergenerational co-residence). Secondly, a possible explanation for the increase in observed connectedness in this study might be increased connectedness by smartphone use. Therefore, controlling for smartphone use might show differences in observed connectedness. Thus, further investigation is required to understand the relationship between smartphone use and loneliness, mediated by connectedness [61, 62]. Thirdly, further research should also investigate differences in social connections and how they might affect wellbeing during the COVID-19 pandemic. For example, it could be hypothesized that people who maintained low connectedness levels before the pandemic would be less affected by the lockdown than someone whose connectedness decreased after the outbreak due to less participation in social activities. That is, contact loss may negatively impact the quality of life [7, 63]. Finally, the results indicated an increase in intergenerational co-residence, and people living alone may be an object of detailed future research due to a possible significant negative impact on the feeling of loneliness compared to people living with others [64].

Overall, these findings have confirmed that older adults have social and psychological resources that allow them to face adversity, such as the COVID-19 pandemic, and have provided important insights into possible future research directions when investigating the impact of COVID-19 on older adults’ health. Furthermore, important resources that enable older adults to better cope during anxious times can guide the use of effective strategies to ensure older adults’ health is not negatively affected by adverse situations, such as COVID-19.

## Supplementary Information

**Additional file 1: Table S1.** Pair correlations between variables during the COVID-19 outbreak (follow-up sample).

## Data Availability

Data and Software Code (in Stata 14.0 Software) are available on request to mherrepo@uc.cl.

## References

[1] INE. Estimaciones y proyecciones de la población de Chile 2002–2035. Totales regionales, población urbana y rural. Instituto Nacional de Estadísticas de Chile; 2019. Available from: https://www.ine.cl/docs/default-source/proyecciones-de-poblacion/publicaciones-y-anuarios/base-2017/ine_estimaciones-y-proyecciones-2002-2035_base-2017_reg_%C3%A1rea_s%C3%ADntesis.pdf?sfvrsn=aaeb88e7_5

[2] Villalobos Dintrans P, Browne J, Madero-Cabib I (2020). It is not just mortality: a call from chile for comprehensive covid-19 policy responses among older people. J Gerontol Ser B.

[3] NCHS. Weekly updates by select demographic and geographic characteristics provisional death counts for Coronavirus Disease 2019 (COVID-19): National Center for Health Statistics. Centers for Disease Control and Prevention; 2020. Updated: September 30, 2020

[4] Armitage R, Nellums LB (2020). COVID-19 and the consequences of isolating the elderly. Lancet Public Health.

[5] Tyrrell CJ, Williams KN (2020). The paradox of social distancing: implications for older adults in the context of COVID-19. Psychol Trauma Theory Res Pract Policy.

[6] Whitehead BR, Torossian E (2020). Older adults’ experience of the covid-19 pandemic: a mixed-methods analysis of stresses and joys. Gerontologist.

[7] Arpino B, Pasqualini M, Bordone V, Solé-Auró A (2020). Older people’s non-physical contacts and depression during the covid-19 lockdown. Gerontologist.

[8] van Tilburg T, Steinmetz S, Stolte E, van der Roest H, de Vries D (2020). Loneliness and mental health during the covid-19 pandemic: a study among dutch older adults. J Gerontol B Psychol Sci Soc Sci.

[9] Krendl AC, Perry BL (2020). The impact of sheltering-in-place during the COVID-19 pandemic on older adults’ social and mental wellbeing. J Gerontol B Psychol Sci Soc Sci.

[10] Kivi M, Hansson I, Bjälkebring P (2020). Up and about: Older adults’ wellbeing during the COVID-19 pandemic in a Swedish longitudinal study. J Gerontol Ser B.

[11] García-Soler Á, Castejón P, Marsillas S. Ageism and COVID-19: A study of social inequality through opinions and attitudes about older people in the coronavirus crisis in Spain. LTCcovid.org, International Long-Term Care Policy Network, CPEC-LSE, 12 June 2020.; 2020. Available from: https://ltccovid.org/wp-content/uploads/2020/08/COVID-and-ageism-an-attitudes-survey-in-Spain.pdf

[12] Previtali F, Allen LD, Varlamova M (2020). Not only virus spread: the diffusion of ageism during the outbreak of COVID-19. J Aging Soc Policy.

[13] Rahman A, Jahan Y (2020). Defining a ‘Risk Group’ and ageism in the era of COVID-19. J Loss Trauma.

[14] Reynolds L (2020). The COVID-19 pandemic exposes limited understanding of ageism. J Aging Soc Policy.

[15] Vervaecke D, Meisner BA (2020). Caremongering and Assumptions of Need: The Spread of Compassionate Ageism During COVID-19. Gerontologist.

[16] Ayalon L, Chasteen A, Diehl M, Levy BR, Neupert SD, Rothermund K (2020). Aging in times of the COVID-19 pandemic: avoiding ageism and fostering intergenerational solidarity. J Gerontol Ser B.

[17] Monahan C, Macdonald J, Lytle A, Apriceno M, Levy SR (2020). COVID-19 and ageism: how positive and negative responses impact older adults and society. Am Psychol.

[18] Tripathy S (2020). The COVID-19 pandemic and the elderly patient: review of current literature and knowledgebase. J Geriatr Care Res.

[19] González-Sanguino C, Ausín B, Castellanos MÁ, Saiz J, López-Gómez A, Ugidos C (2020). Mental health consequences during the initial stage of the 2020 Coronavirus pandemic (COVID-19) in Spain. Brain Behav Immun.

[20] Talevi D, Socci V, Carai M, Carnaghi G, Faleri S, Trebbi E (2020). Mental health outcomes of the COViD-19 pandemic. Riv Psichiatr.

[21] Zhang J, Lu H, Zeng H, Zhang S, Du Q, Jiang T (2020). The differential psychological distress of populations affected by the COVID-19 pandemic. Brain Behav Immun.

[22] Cugmas M, Ferligoj A, Kogovšek T, Batagelj Z. The social support networks of elderly people in Slovenia during the Covid-19 pandemic. SocArXiv. 2020. 10.31235/osf.io/uat4e. 10.1371/journal.pone.0247993 PMC7928497 33657172

[23] López J, Perez-Rojo G, Noriega C, Carretero I, Velasco C, Martinez-Huertas JA (2020). Psychological wellbeing among older adults during the COVID-19 outbreak: A comparative study of the young–old and the old–old adults. Int Psychogeriatr.

[24] Röhr S, Reininghaus U, Riedel-Heller SG (2020). Mental wellbeing in the German old age population largely unaltered during COVID-19 lockdown: results of a representative survey. BMC Geriatr.

[25] Cheng TC, Kim S, Koh K (2020). The impact of COVID-19 on subjective wellbeing: evidence from Singapore. In 13702: IZA Discussion Paper.

[26] Borson S, Scanlan J, Brush M, Vitaliano P, Dokmak A (2000). The mini-cog: a cognitive ‘vital signs’ measure for dementia screening in multi-lingual elderly. Int J Geriatr Psychiatry.

[27] Borson S, Scanlan JM, Chen P, Ganguli M (2003). The mini-cog as a screen for dementia: validation in a population-based sample. J Am Geriatr Soc.

[28] UC, Caja-Los-Andes (2020). Chile y sus mayores. Quinta Encuesta Nacional de Calidad de Vida en la Vejez 2019.

[29] Kroenke K, Spitzer RL, Williams JB (2001). The PHQ-9: validity of a brief depression severity measure. J Gen Intern Med.

[30] Saldivia S, Aslan J, Cova F, Vicente B, Inostroza C, Rincón P (2019). Propiedades psicométricas del PHQ-9 (Patient Health Questionnaire) en centros de atención primaria de Chile. Rev Médica Chile.

[31] Byrne GJ, Pachana NA (2011). Development and validation of a short form of the geriatric anxiety inventory-the GAI-SF. Int Psychogeriatr.

[32] Sinclair VG, Wallston KA (2004). The development and psychometric evaluation of the brief resilient coping scale. Assessment..

[33] Tomás J, Meléndez J, Sancho P, Mayordomo T (2012). Adaptation and initial validation of the BRCS in an elderly Spanish sample. Eur J Psychol Assess.

[34] Lubben J, Blozik E, Gillmann G, Iliffe S, von Renteln KW, Beck JC (2006). Performance of an abbreviated version of the Lubben social network scale among three European community-dwelling older adult populations. The Gerontologist.

[35] Hughes ME, Waite LJ, Hawkley LC, Cacioppo JT (2004). A short scale for measuring loneliness in large surveys: results from two population-based studies. Res Aging.

[36] Shankar A, Rafnsso S, Steptoe A (2015). Longitudinal associations between social connections and subjective wellbeing in the English longitudinal study of ageing. Psychol Health.

[37] Jeste DV, Savla GN, Thompson WK, Vahia IV, Glorioso DK, Martin AS (2013). Association between older age and more successful aging: critical role of resilience and depression. Am J Psychiatry.

[38] Smith JL, Hollinger-Smith L (2015). Savoring, resilience, and psychological wellbeing in older adults. Aging Ment Health.

[39] Toukhsati S, Jovanovic A, Dehghani S, Tran T, Tran A, Hare D (2016). Low psychological resilience is associated with depression in patients with cardiovascular disease. Eur J Cardiovasc Nurs.

[40] Edward K (2013). Chronic illness and wellbeing: using nursing practice to foster resilience as resistance. Br J Nurs.

[41] Manning LK, Carr DC, Kail BL (2016). Do higher levels of resilience buffer the deleterious impact of chronic illness on disability in later life?. The Gerontologist.

[42] Liu J-C, Chang L-Y, Wu S-Y, Tsai P-S (2015). Resilience mediates the relationship between depression and psychological health status in patients with heart failure: a cross-sectional study. Int J Nurs Stud.

[43] Zhao X, Zhang D, Wu M, Yang Y, Xie H, Li Y (2018). Loneliness and depression symptoms among the elderly in nursing homes: a moderated mediation model of resilience and social support. Psychiatry Res.

[44] MacLeod S, Musich S, Hawkins K, Alsgaard K, Wicker ER (2016). The impact of resilience among older adults. Geriatr Nur (Lond).

[45] Stewart DE, Yuen T (2011). A systematic review of resilience in the physically ill. Psychosomatics..

[46] Wells M (2012). Resilience in older adults living in rural, suburban, and urban areas. Online J Rural Nurs Health Care.

[47] Xie Y, Ma M, Wu W, Zhang Y, Zhang Y, Tan X (2020). Dose–response relationship between intergenerational contact frequency and depressive symptoms amongst elderly Chinese parents: a cross-sectional study. BMC Geriatr.

[48] Beneito-Montagut R, Cassián-Yde N, Begueria A (2018). What do we know about the relationship between internet-mediated interaction and social isolation and loneliness in later life?. Qual Ageing Older Adults.

[49] Herrera MS, Barros C, Fernández MB (2011). Predictors of quality of life in old age: a multivariate study in Chile. J Popul Ageing.

[50] Luchetti M, Lee JH, Aschwanden D, Sesker A, Strickhouser JE, Terracciano A (2020). The trajectory of loneliness in response to COVID-19. Am Psychol.

[51] Klaiber P, Wen JH, DeLongis A, Sin NL (2020). The ups and downs of daily life during COVID-19: Age differences in affect, stress, and positive events. J Gerontol B Psychol Sci Soc Sci.

[52] Rodríguez-Rey R, Garrido-Hernansaiz H, Collado S (2020). Psychological impact and associated factors during the initial stage of the coronavirus (COVID-19) pandemic among the general population in Spain. Front Psychol.

[53] Lind M, Bluck S, McAdams DP (2020). More Vulnerable? The Life Story Approach Highlights Older People's Potential for Strength During the Pandemic. J Gerontol Ser B.

[54] Herrera MS, Fernández MB (2020). Stressful events in old age: who are most exposed and who are most likely to overcome them. Gerontol Geriatr Med.

[55] Meda N, Slongo I (2020). Caution when linking COVID-19 to mental health consequences. Brain Behav Immun.

[56] Keeter S, Hatley N, Kennedy C, Lau A (2017). What low response rates mean for telephone surveys. Pew Res Cent.

[57] Herrera MS, Devilat D, Fernández MB, Elgueta R (2020). Does the selective attrition of a panel survey of older people affect the multivariate estimations of subjective wellbeing?. Qual Life Res.

[58] Pearman A, Hughes ML, Smith EL, Neupert SD (2020). Age Differences in Risk and Resilience Factors in COVID-19-Related Stress. J Gerontol Ser B.

[59] Chen S, Bonanno GA (2020). Psychological adjustment during the global outbreak of COVID-19: a resilience perspective. Psychol Trauma Theory Res Pract Policy.

[60] Vinkers CH, Van Amelsvoort T, Bisson JI, Branchi I, Cryan JF, Domschke K (2020). Stress resilience during the coronavirus pandemic. Eur Neuropsychopharmacol J Eur Coll Neuropsychopharmacol.

[61] Beaunoyer E, Dupéré S, Guitton MJ (2020). COVID-19 and digital inequalities: Reciprocal impacts and mitigation strategies. Comput Hum Behav.

[62] Chen AT, Ge S, Cho S, Teng AK, Chu F, Demiris G, et al. Reactions to COVID-19, information and technology use, and social connectedness among older adults with pre-frailty and frailty. Geriatr Nur (Lond). 2020. 10.1016/j.gerinurse.2020.08.001. 10.1016/j.gerinurse.2020.08.001 PMC7416746 32863038

[63] Roy J, Jain R, Golamari R, Vunnam R, Sahu N (2020). COVID-19 in the geriatric population. Int J Geriatr Psychiatry.

[64] Routasalo P, Pitkala KH (2003). Loneliness among older people. Rev Clin Gerontol.

