# Supplementary material for: A longitudinal study monitoring the quality of life in a national cohort of older adults in Chile before and during the COVID-19 outbreak
Source: BMC Geriatr. 2021 Feb 26;21:143. doi: 10.1186/s12877-021-02110-3 (PMC7908522; doi:10.1186/s12877-021-02110-3)
Supplement: Supplementary file 1 — Additional file 1: Table S1. Pair correlations between variables during the COVID-19 outbreak (follow-up sample). [file 12877_2021_2110_MOESM1_ESM.docx]

**A longitudinal study monitoring the quality of life in a national cohort of older adults in Chile before and during the COVID-19 outbreak**

M.Soledad Herrera, PhD ^1^ (corresponding – [mherrepo@uc.cl](mailto:mherrepo@uc.cl) )

Raúl Elgueta, PhD ^2^

M.Beatriz Fernández, PhD ^1^

Claudia Giacoman, PhD ^1^

Daniella Leal, MSc ^1^

Pío Marshall, MSc ^1^

Miriam Rubio, Msc ^3^

Felipe Bustamante ^1^

^1^ Instituto de Sociología (Department of Sociology), Pontificia Universidad Católica de Chile.

^2^ Instituto de Estudios Avanzados (Institute of Advanced Studies), Universidad de Santiago de Chile.

^3^  Escuela de Enfermería (Nursing School), Pontificia Universidad Católica de Chile.

**Table S1**

*Pair correlations between variables during the COVID-19 outbreak (follow-up sample)*

|  | 1 | 2 | 3 | 4 | 5 | 6 | 7 | 8 | 9 | 10 | 11 | 12 |
| --- | --- | --- | --- | --- | --- | --- | --- | --- | --- | --- | --- | --- |
| 1. PHQ-9 for depressive symptoms (0-27) | 1.0000 |  |  |  |  |  |  |  |  |  |  |  |
| 2. Geriatric Anxiety Inventory GAI-SF (0-5) | 0.5145* | 1.0000 |  |  |  |  |  |  |  |  |  |  |
| 3. Memory problems (0-1) | 0.3167* | 0.2207* | 1.0000 |  |  |  |  |  |  |  |  |  |
| 4. Stomach or bowel problems (0-1) | 0.3187* | 0.2260* | 0.1330* | 1.0000 |  |  |  |  |  |  |  |  |
| 5. Brief Resilient Coping Scale (0-16) | -0.2023* | -0.1037* | -0.1524* | -0.0839* | 1.0000 |  |  |  |  |  |  |  |
| 6. Brief Lubben Social Network Scale (0-30) | -0.1269* | -0.1179* | -0.0331 | -0.0320 | 0.1359* | 1.0000 |  |  |  |  |  |  |
| 7. UCLA-3 Loneliness Scale (0-6) | 0.4857* | 0.4072* | 0.2052* | 0.2542* | -0.1312* | -0.26114* | 1.0000 |  |  |  |  |  |
| 8. Multigeneration household (with children and / or grandchildren) | -0.0309 | -0.0218 | -0.0105 | -0.0349 | -0.0123 | -0.0247 | -0.0756* | 1.0000 |  |  |  |  |
| 9. Woman | 0.0504 | 0.0613 | -0.0108 | 0.0895* | 0.0907* | -0.0151 | 0.0193 | 0.0088 | 1.0000 |  |  |  |
| 10. Age (60-92 years old) | -0.0860* | -0.1218* | 0.0253 | -0.0322 | -0.0328 | -0.0415 | -0.0927* | 0.0317 | 0.0268 | 1.0000 |  |  |
| 11. Education | -0.0388 | 0.0193 | -0.0537 | -0.0173 | 0.0310 | 0.1072* | -.0377 | -0.0861* | -1.1211* | -0.2228* | 1.0000 |  |
| 12. Sufficient income | -0.1338* | -0.1714* | -0.0874* | -0.0765* | 0.0367 | 0.0842* | -0.0871* | -0.1174* | -0.0419 | 0.0999* | 0.0804* | 1.0000 |
| 13. Smartphone user | -0.0065 | 0.0906* | -.0854* | 0.1407* | 0.1098* | 0.1996* | -0.0102 | -0.0728 | 0.0087 | -0.3610* | 0.2679 | 0.0499 |

N between 708 and 721.

**p* < .05.
